# Psychosocial Job Characteristics and Job Strain Among Nurses in Urban Primary Healthcare Centers in Vietnam: A Multicenter Cross-Sectional Study

**DOI:** 10.3390/ijerph23080962

**Published:** 2026-07-25

**Authors:** Mi Phan Thanh Tra, Xuan Thanh Phan

**Affiliations:** Faculty of Public Health, University of Medicine and Pharmacy at Ho Chi Minh City, 217 Hong Bang, Cho Lon Ward, Ho Chi Minh 700000, Vietnam; pttmi.ncs21@ump.edu.vn

**Keywords:** psychosocial job characteristics, job strain, nurses, primary healthcare, demand–control–support model, Vietnam

## Abstract

**Highlights:**

**Public health relevance—How does this work relate to a public health issue?**

- Psychosocial working conditions are important in primary healthcare.
- Evidence from urban primary healthcare centers in Vietnam remains limited.

**Public health significance—Why is this work of significance to public health?**

- Low-strain and active jobs predominated.
- Job strain was associated with several sociodemographic and work-related factors.

**Public health implications—What are the key implications or messages for practitioners, policy makers and/or researchers in public health?**

- The findings provide context-specific evidence.
- Future longitudinal and intervention research is needed.

**Abstract:**

Psychosocial job characteristics are important components of the work environment and are closely associated with occupational health, particularly among nurses working in demanding healthcare settings. However, evidence from primary healthcare settings in low- and middle-income countries remains limited. This study aimed to describe psychosocial job characteristics and job strain among nurses in urban primary healthcare centers in Vietnam and to examine factors associated with psychosocial job characteristics and job strain. A multicenter cross-sectional study was conducted among 768 nurses across four district-level healthcare centers in Ho Chi Minh City, Vietnam, using a structured questionnaire including the validated Vietnamese Job Content Questionnaire. Nurses reported moderate-to-high job demands, high social support, and limited decision latitude. Most participants reported low strain (45.6%) or active jobs (42.4%), while fewer were in the high-strain (6.3%) and passive (5.7%) categories. Sociodemographic and work-related factors were significantly associated with job strain (*p* < 0.001). Although the prevalence of high strain was relatively low, differences between perceived job demands and specific dimensions of decision latitude were observed. These findings highlight the importance of job control, workload, and workplace support as factors associated with psychosocial job characteristics and job strain and may help inform future workplace interventions and longitudinal research.

## 1. Introduction

Psychosocial job characteristics are important components of the work environment and are closely associated with occupational health and well-being, particularly among nurses working in complex and demanding clinical settings [1,2]. The demand–control–support model conceptualizes job strain as a condition arising from the interaction between high job demands, low decision latitude, and inadequate social support [2,3]. Within this framework, nurses are especially vulnerable due to their continuous exposure to patient care, time pressure, and organizational constraints [2,3]. Globally, increasing healthcare demands and workforce shortages have intensified these psychosocial stressors, placing nurses at elevated risk of burnout, psychological distress, and reduced quality of life [4,5]. Recent evidence indicates that burnout, psychological distress, and other work-related mental health problems affect a substantial proportion of nurses worldwide, underscoring the growing importance of improving psychosocial working conditions in healthcare settings [4,5]. However, empirical evidence consistently shows that imbalances between job demands and available resources are common in nursing settings [1]. Therefore, examining psychosocial job characteristics provides a critical lens for understanding workforce sustainability in modern health systems.

The consequences of psychosocial job characteristics and job strain extend beyond individual health outcomes and have direct implications for clinical performance and patient care. Nurses experiencing high job strain are more likely to report decreased work efficiency, emotional exhaustion, and reduced engagement in clinical tasks [6,7]. Importantly, these factors have been associated with increased risk of medical errors, compromised patient safety, and lower quality of care [8,9]. Job strain may impair cognitive functioning, decision-making capacity, and communication, all of which are essential for effective clinical practice [10]. Furthermore, adverse psychosocial work environments can weaken teamwork and reduce coordination across healthcare providers [1]. Previous studies have suggested that greater decision latitude and stronger workplace social support are associated with more favorable psychosocial work outcomes [3].

Despite extensive research on job strain among nurses [1], several important gaps remain. First, the majority of existing studies have been conducted in high-income countries [5,11], limiting the applicability of findings to healthcare systems with different organizational and resource contexts [5]. Second, many studies have focused on isolated dimensions of occupational stress rather than applying comprehensive theoretical models such as the demand–control–support framework [2]. Third, although previous studies have investigated psychosocial job characteristics among nurses in primary healthcare settings [12], evidence from low- and middle-income countries, particularly Vietnam, remains limited. Moreover, nurses in primary healthcare settings often work under different conditions compared with those in hospital environments [13]. These gaps highlight the need for context-specific studies to better understand psychosocial job characteristics and factors associated with job strain among nurses working in primary healthcare settings in Vietnam, thereby providing evidence to inform future longitudinal studies and intervention research [11,12].

In Vietnam, rapid health system expansion, increasing patient demand, and workforce constraints have created substantial pressure on healthcare workers, particularly nurses in primary healthcare settings [14,15]. These professionals often face prolonged working hours, high workloads, and competing responsibilities between work and family roles [15]. However, empirical evidence on psychosocial job characteristics and job strain in this context remains limited. Therefore, this study aims to describe psychosocial job characteristics and job strain among nurses in urban primary healthcare centers in Vietnam. In addition, the study seeks to examine sociodemographic and work-related factors associated with job strain. The findings are expected to provide evidence on psychosocial job characteristics and factors associated with job strain and may help inform future workplace policies and research in primary healthcare settings.

## 2. Materials and Methods

### 2.1. Study Design and Participants

This quantitative study employed a multicenter cross-sectional design conducted in urban primary healthcare centers in Ho Chi Minh City, Vietnam. The target population included all nurses working at district-level healthcare centers, while the study population comprised nurses who were actively working in selected healthcare centers. The study was conducted in December 2025 across four healthcare centers, including Tan Phu District Health Center, District 6 Health Center, Thu Duc District Health Center, and Binh Chanh District Health Center. This study used a census sampling approach, whereby all eligible nurses working at the four selected healthcare centers were invited to participate through a voluntary online survey. Eligible participants were those who were currently employed as nurses at the selected centers. A survey link was distributed to all eligible nurses (approximately 1000 nurses). A total of 783 nurses responded to the survey. After excluding 15 incomplete questionnaires, 768 completed questionnaires were included in the final analysis.

### 2.2. Study Procedures

Data were collected using a structured, self-administered questionnaire distributed to participants via an online platform. The entire study protocol, including the online recruitment process, participant information sheet, and implied informed consent through voluntary completion and submission of the anonymous online questionnaire, was reviewed and approved by the Ethics Committee for Biomedical Research at the University of Medicine and Pharmacy at Ho Chi Minh City (Approval No. 806/HĐĐĐ/ĐHYD). Before accessing the questionnaire, participants were provided with an electronic participant information sheet explaining the purpose of the study, confidentiality measures, voluntary participation, and their rights, including that they could withdraw at any time without any consequences. The information sheet also clearly stated that completion and submission of the online questionnaire would be considered as implied informed consent to participate in the study. Completed questionnaires were reviewed for completeness and consistency, and invalid or incomplete responses were excluded. Data were then coded and entered into a database for analysis.

### 2.3. Measurements

The questionnaire included three main components. The first component was about sociodemographic characteristics such as age, sex, marital status, caregiving responsibilities, housing status, and income. The second component captured work-related characteristics, including educational level, department, years of experience, weekly working hours, and additional responsibilities. The third component assessed psychosocial job characteristics using the Karasek Job Content Questionnaire (JCQ) [2,16,17]. The JCQ is widely recognized as a standard tool for measuring job strain and comprises 33 items covering three domains: job demand, decision latitude, and social support at work [16]. The Vietnamese version of the JCQ had been previously translated and validated [16,17]. The internal consistency of the JCQ domains in the present study was good, with Cronbach’s alpha coefficients of 0.937 for job demand, 0.801 for decision latitude, and 0.892 for social support.

The JCQ items were scored on a 4-point Likert scale ranging from 1 (“strongly disagree”) to 4 (“strongly agree”). Domain scores were calculated by summing the relevant items without weighting. Job demand was calculated from 8 items, with a possible score range of 8–32. Decision latitude was calculated from 17 items, with a possible score range of 17–68. Social support was calculated from 8 items, with a possible score range of 8–32. For descriptive presentation, job demand and social support scores were categorized into four levels, namely very low, low, high, and very high, using the following ranges: 8, 9–16, 17–24, and 25–32, respectively. Decision latitude was categorized using the following ranges: 17, 18–34, 35–51, and 52–68, respectively.

Job strain was classified by combining job demand and decision latitude according to the demand–control model. Job demand was dichotomized as low (≤16) and high (>16), while decision latitude was dichotomized as low (≤34) and high (>34). Four categories were then created: high strain (high demand/low decision latitude), passive job (low demand/low decision latitude), active job (high demand/high decision latitude), and low strain (low demand/high decision latitude).

### 2.4. Data Analysis

Descriptive statistics were used to summarize participants’ characteristics and psychosocial job factors, with categorical variables presented as frequencies and percentages and continuous variables as means and standard deviations. For a descriptive presentation of individual JCQ items, the original four response categories were collapsed into two groups (strongly disagree/disagree and agree/strongly agree) to facilitate interpretation of participants’ responses. To preserve the original quantitative information, the mean and standard deviation of each item based on the original four-point Likert responses are also presented. This dichotomization was applied solely for descriptive presentation and did not affect the calculation of JCQ domain scores or any subsequent statistical analyses, which were based on the original four-point item scores. Moreover, given the descriptive and exploratory nature of the study, analyses were primarily based on descriptive statistics and conventional bivariate tests without further multivariable modeling. Chi-square tests were used to compare categorical variables. For continuous variables, independent-sample *t*-tests were used to compare two groups, while one-way analysis of variance (ANOVA) was used to compare more than two groups, as appropriate. A *p*-value of less than 0.05 was considered statistically significant.

## 3. Results

### 3.1. Participants’ Characteristics

Among 768 nurses, the majority were female (72.5%) and aged between 31 and 50 years (86.5%) (Table 1). Most participants had a bachelor’s degree or higher (87.9%) and were married (68.9%). A large proportion reported renting their accommodation (69.3%) and having a monthly income between 7 and 14 million VND (77.6%). Additionally, most nurses were responsible for caregiving duties, including caring for children under five years (66.1%) and elderly or disabled family members (79.9%). Nearly half had more than nine years of experience (44.4%), and the majority worked in clinical departments (69.0%), with 41.5% reporting more than 48 working hours per week.

### 3.2. Psychosocial Job Characteristics and Job Strain

**Figure 1:**
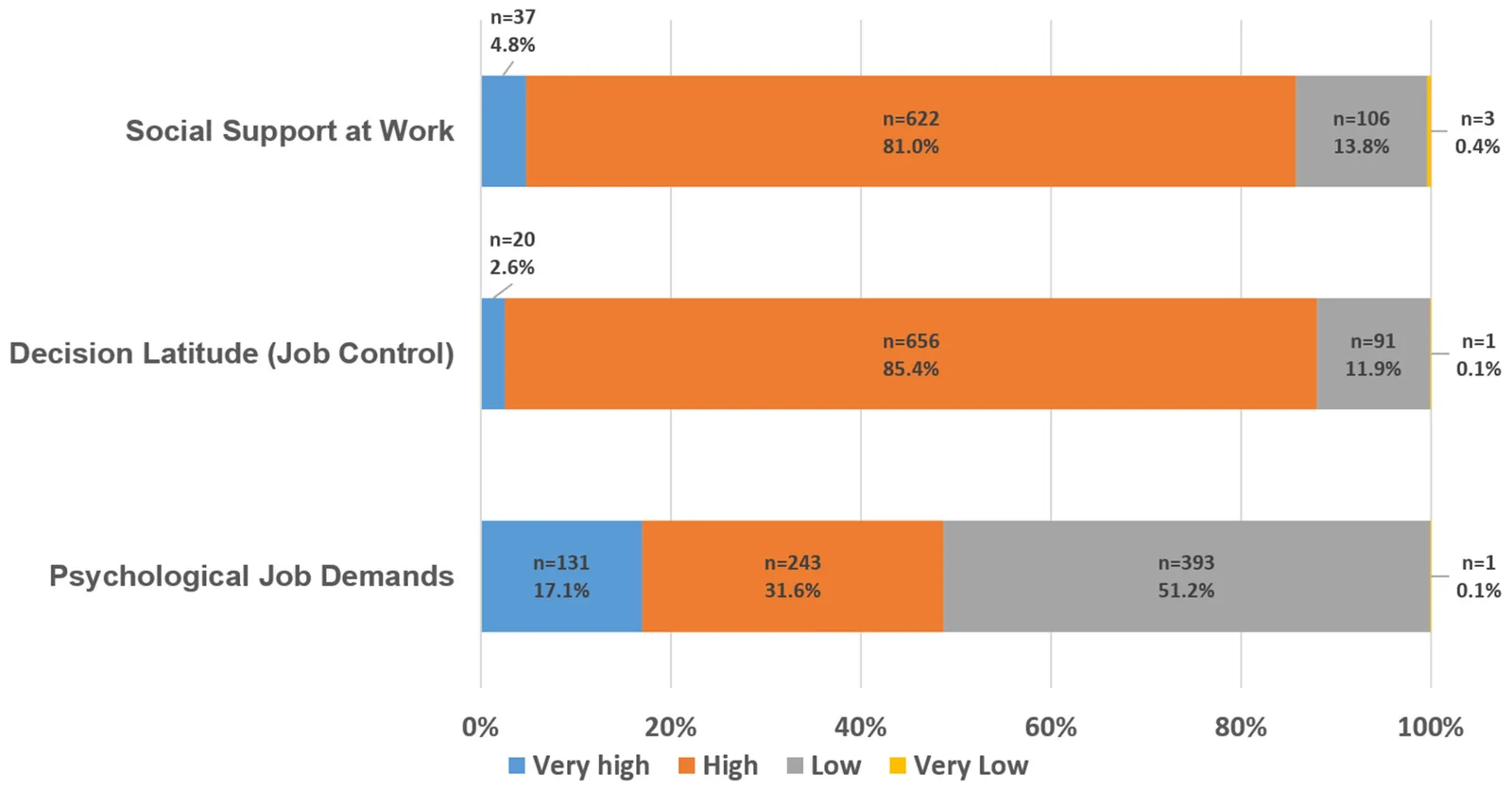
Psychosocial job characteristics based on the Job Content Questionnaire among nurses from primary healthcare centers.

**Figure 2:**
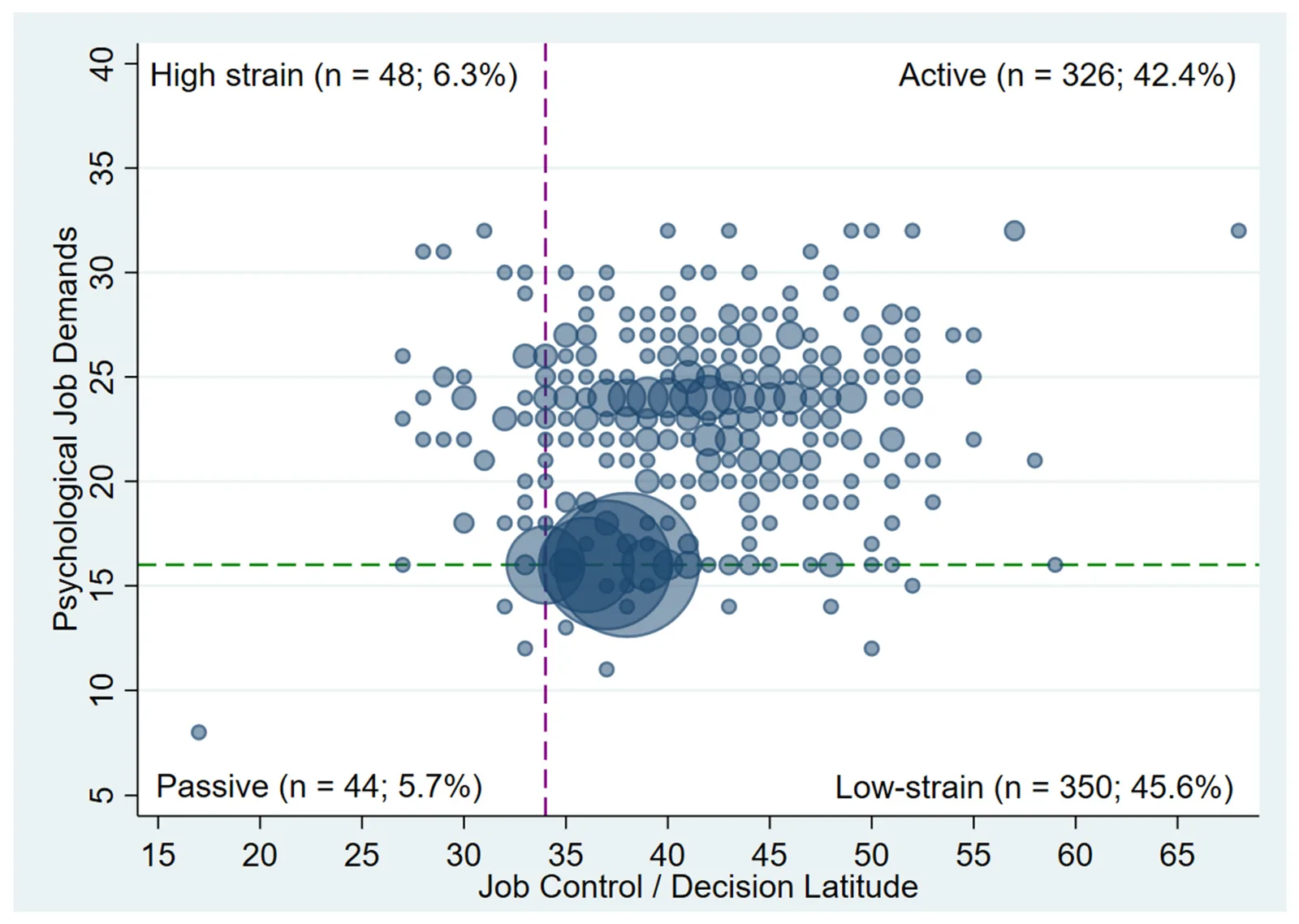
Job strain among nurses in primary healthcare centers. Note: Larger bubbles indicate higher frequencies (number of participants).

Psychosocial job characteristics are presented in Table 2, showing that the mean scores were highest for social support, followed by job demand and decision latitude. While job demand items were relatively balanced between agreement and disagreement, indicators of decision latitude consistently showed lower agreement levels, suggesting restricted autonomy among nurses. In contrast, most participants reported favorable social support conditions, particularly in supervisor relationships and workplace communication. As illustrated in Figure 1, most nurses reported moderate-to-high levels of job demands and decision latitude, with the majority classified as low or high rather than extreme categories, while social support was predominantly high. According to the job strain model (Figure 2), the majority of nurses reported either low strain (45.6%) or active jobs (42.4%), whereas relatively small proportions were in the high-strain (6.3%) and passive (5.7%) categories.

### 3.3. Factors Associated with Psychosocial Job Characteristics and Job Strain

Significant associations were observed between sociodemographic characteristics and job strain distribution (*p* < 0.001) (Table 3). Female nurses were more likely to experience low strain (57.1%), whereas male nurses more frequently reported active jobs (71.1%) and high strain (10.0%). Age also showed a clear gradient association, with younger nurses more likely to report low strain, while older nurses were more likely to be in the active group. Marital status, education level, housing status, income, and caregiving responsibilities were all significantly associated with job strain categories. Notably, individuals with lower income and caregiving responsibilities were more likely to experience passive or high-strain conditions.

Work-related characteristics were strongly associated with job strain (*p* < 0.001) (Table 4). Nurses with longer working hours (>48 h per week) and those holding managerial or additional positions were more likely to report active or high-strain jobs. In contrast, nursesworking ≤40 h per week were predominantly classified as low strain (86.0%). Department type also played a significant role, with nurses in clinical departments more likely to report low strain, whereas those in administrative and paraclinical departments were more likely to fall into the active or high-strain categories. Length of employment was also associated with job strain, with more experienced nurses tending toward active job profiles.

## 4. Discussion

This study is among the first to provide a comprehensive assessment of psychosocial job characteristics and job strain among nurses in urban primary healthcare settings in Vietnam. Overall, the findings indicate that nurses experienced moderate-to-high job demands, relatively favorable social support, and limited decision latitude. When classified according to the job strain model, the majority of participants were distributed in the low-strain and active job categories, while only a small proportion reported high strain. Significant associations were observed between psychosocial job components, job strain categories, and a range of sociodemographic and work-related factors, indicating that nurses’ psychosocial working conditions varied according to individual and organizational characteristics.

### 4.1. Psychosocial Job Characteristics

Our findings reveal a distinct imbalance between job demands, decision latitude, and social support. Consistent with the literature, job demand levels were generally moderate to high [16,18], reflecting the inherently demanding nature of nursing work, including workload intensity, time pressure, and continuous patient care [1,19]. In contrast, decision latitude was consistently lower [20], indicating limited autonomy and restricted control over work processes [21]. This imbalance between high demand and low control represents a key mechanism underlying occupational stress [22]. Notably, in our study, social support was relatively high, indicating positive relationships with colleagues and supervisors. Previous studies have highlighted the protective role of social support in mitigating the negative effects of high job demands [23]. Therefore, while nurses operate in demanding environments, supportive organizational contexts may partially buffer adverse psychosocial conditions. However, improving decision latitude remains critical to achieving a more balanced and sustainable work environment [1].

### 4.2. Job Strain

When examined using the job strain classification, the overall prevalence of high job strain in our study was relatively low and consistent with previous research conducted in Vietnam using the JCQ instrument [16,17]. However, the majority of nurses were classified into low-strain and active job categories. The relatively high proportion of nurses classified in the active job category may also reflect the organizational characteristics of urban primary healthcare centers in Vietnam. Compared with hospital settings, nurses in primary healthcare often undertake diverse responsibilities, including preventive care, chronic disease management, health promotion, and community-based healthcare services [14,15]. These roles may require high job demands while providing greater opportunities for professional decision-making and autonomy, which may partly explain the relatively high proportion of nurses classified in the active job category. Because healthcare organizations, nursing roles, and local practice requirements differ across settings and countries, such comparisons should be interpreted with caution. While the active job category is generally considered less detrimental than high strain, previous studies have suggested that prolonged exposure to high job demands may be associated with fatigue and burnout over time [3]. The relatively low proportion of passive jobs suggests that most nurses were actively engaged in their work, although work engagement and productivity were not directly assessed in the present study [24]. Nevertheless, the small but important proportion of high-strain workers remains a concern due to its association with adverse health and performance outcomes. These findings highlight the importance of interpreting job strain distribution holistically rather than focusing solely on high strain prevalence [25].

### 4.3. Factors Associated with Psychosocial Job Characteristics

In our study, several sociodemographic and work-related factors were significantly associated with job demands, decision latitude, and social support. Specifically, factors such as age, sex, income, and caregiving responsibilities were linked to variations in perceived job demands and social support. Nurses with greater family responsibilities, particularly caregiving for children or elderly relatives, may experience higher perceived demands due to competing roles [26,27]. Work-related characteristics, including working hours, department type, and managerial roles, were strongly associated with job demands and decision latitude. These findings are consistent with previous studies demonstrating that longer working hours, managerial responsibilities, and both personal and organizational factors influence psychosocial working conditions among nurses [22,28]. Our findings suggest that psychosocial job characteristics were associated with both structural and contextual factors. Future studies are warranted to further investigate these relationships and inform the development of workplace interventions.

### 4.4. Factors Associated with Job Strain

Similar patterns of association were observed when psychosocial working conditions were analyzed using job strain categories. Sociodemographic factors, including sex, age, marital status, educational level, and income, were significantly associated with the distribution of job strain categories. Previous studies have shown that differences in job strain between male and female nurses may reflect variations in role expectations and coping mechanisms [29]. Moreover, family-related factors, including caregiving responsibilities, were also associated with a higher likelihood of passive or high-strain conditions. Similar to previous studies, work-related characteristics, particularly long working hours [30] and managerial responsibilities [31], were associated with an increased likelihood of being classified in active or high-strain groups [32]. These findings suggest that job strain is not only a function of workplace conditions but also influenced by broader social and family contexts [27].

### 4.5. Implications of the Study

The findings may have implications for healthcare management and policy in urban primary healthcare settings similar to those included in this study. First, future workplace interventions may consider enhancing nurses’ autonomy and involvement in decision-making processes, as previous studies have suggested that greater decision latitude may help reduce job strain [22,33]. Second, maintaining supportive workplace environments may be beneficial. Previous studies have suggested that social support is associated with more favorable psychosocial working conditions, although these outcomes were not directly assessed in the present study [23,34]. Third, future workplace strategies may consider workload management, including optimizing staffing levels and working hours [35]. The effectiveness of such strategies should be evaluated in future interventions and longitudinal studies. Additionally, targeted interventions should focus on vulnerable groups, such as nurses with significant caregiving responsibilities or those working extended hours, to alleviate work-family conflict and associated stress [26,36]. These strategies aim to balance job demands with available resources, including decision latitude and workplace support.

### 4.6. Study Limitations

Several limitations should be considered when interpreting the findings of this study. First, the cross-sectional design limits the ability to establish causal relationships. Second, the use of self-reported data may introduce reporting bias and social desirability bias. Furthermore, as a quantitative study, the findings may not fully capture participants’ experiences or the contextual factors underlying psychosocial job characteristics and job strain. Qualitative or mixed-methods studies may provide deeper insights into these issues. Third, the study was conducted in selected urban primary healthcare centers in Ho Chi Minh City. Therefore, the findings may not be generalizable to rural primary healthcare settings, hospitals, private healthcare facilities, or other regions of Vietnam. The results should be interpreted within the context of the study population. Additionally, although multiple factors were examined, other relevant variables such as organizational culture and leadership style were not included. The absence of multivariable analysis may also limit the ability to identify independent determinants of job strain. Future research should consider longitudinal designs and more advanced analytical approaches.

## 5. Conclusions

This study demonstrates that nurses in urban primary healthcare settings experience an imbalance between moderate-to-high job demands and limited decision latitude, despite relatively strong social support. Although the prevalence of high job strain was low, a substantial proportion of nurses were exposed to potentially stressful work conditions, particularly within active job profiles. Both sociodemographic and work-related factors were significantly associated with psychosocial job characteristics and job strain, highlighting the multifactorial nature of occupational stress. These findings provide evidence on psychosocial job characteristics and factors associated with job strain among nurses in urban primary healthcare settings. They may help inform future longitudinal studies and workplace interventions aimed at improving psychosocial working conditions.

## Figures and Tables

**Table 1:** Characteristics of nurses at primary healthcare centers.

Characteristics	Frequency	Percentage
Sex		
Male	211	27.5
Female	557	72.5
Age (year) *Mean (Standard Deviation)*	40.7	6.9
Age category		
≤30	60	7.8
31–40	340	44.3
41–50	324	42.2
>50	44	5.7
Education level		
Vocational/College diploma	79	10.3
Bachelor’s degree or postgraduate	675	87.9
Other	14	1.8
Marital status		
Married	529	68.9
Single	157	20.4
Other	82	10.7
Housing status		
Own house	152	19.8
Rented house	532	69.3
Other	84	10.9
Monthly income (million VND)		
<7 (~300 USD)	163	21.2
7–14 (300–600 USD)	596	77.6
≥14 (≥600 USD)	9	1.2
Caring for children under 5 years		
No	260	33.9
Yes	508	66.1
Caring for elderly or disabled family members		
No	154	20.1
Yes	614	79.9
Length of employment at healthcare centers (year)		
<3	22	2.9
3–6	117	15.2
7–9	288	37.5
>9	341	44.4
Current working department		
Clinical departments	530	69.0
Paraclinical departments	86	11.2
Administrative departments	134	17.4
Logistics/support departments	18	2.3
Weekly working hours		
≤40	378	49.2
41–48	71	9.2
>48	319	41.5
Holding managerial or additional positions		
No	503	65.5
Yes	265	34.5

**Table 2:** Psychosocial job characteristics among nurses at primary healthcare centers.

JCQ Item	Mean (SD)	Category	
		Strongly Disagree/Disagree	Strongly Agree/Agree
(JD) Heavy workload	2.47 (0.66)	438 (57.0)	330 (43.0)
(JD) Work overload	2.51 (0.71)	445 (57.9)	323 (42.1)
(JD) Insufficient time for tasks	2.49 (0.70)	449 (58.5)	319 (41.5)
(JD) Unreasonable job demands	2.49 (0.71)	458 (59.6)	310 (40.4)
(JD) Prolonged concentration requirement	2.49 (0.71)	455 (59.2)	313 (40.8)
(JD) Frequent work interruptions	2.46 (0.68)	468 (60.9)	300 (39.1)
(JD) Constant busyness	2.45 (0.66)	464 (60.4)	304 (39.6)
(JD) Delays from others	2.45 (0.65)	457 (59.5)	311 (40.5)
Psychological job demand	19.80 (4.56)		
(DL) Decision-making freedom	2.42 (0.64)	487 (63.4)	281 (36.6)
(DL) Work method autonomy	2.35 (0.62)	524 (68.2)	244 (31.8)
(DL) Influence over job matters	2.37 (0.63)	507 (66.0)	261 (34.0)
(DL) Control over task sequence	2.34 (0.61)	535 (69.7)	233 (30.3)
(DL) Flexible working schedule	2.30 (0.61)	546 (71.1)	222 (28.9)
(DL) Ability to leave workstation temporarily	2.35 (0.62)	493 (64.2)	275 (35.8)
(DL) Ability to stop work when desired	2.32 (0.64)	505 (65.8)	263 (34.2)
(DL) Control over work pace	2.12 (0.52)	646 (84.1)	122 (15.9)
(DL) Learning requirement	2.40 (0.66)	415 (54.0)	353 (46.0)
(DL) Low task repetition	2.11 (0.57)	624 (81.3)	144 (18.8)
(DL) Creativity requirement	2.52 (0.66)	340 (44.3)	428 (55.7)
(DL) High skill requirement	2.58 (0.64)	291 (37.9)	477 (62.1)
(DL) Task variety	2.27 (0.59)	538 (70.1)	230 (29.9)
(DL) Opportunities for professional development	2.43 (0.63)	412 (53.6)	356 (46.4)
(DL) Fixed working hours	2.20 (0.53)	598 (77.9)	170 (22.1)
(DL) Break-time autonomy	2.15 (0.54)	615 (80.1)	153 (19.9)
(DL) Leave-time autonomy	2.17 (0.56)	606 (78.9)	162 (21.1)
Decision latitude (job control)	39.40 (5.18)		
(SS) Positive work environment	2.70 (0.59)	231 (30.1)	537 (69.9)
(SS) Low coworker conflict	2.67 (0.60)	232 (30.2)	536 (69.8)
(SS) Coworker support availability	2.67 (0.61)	234 (30.5)	534 (69.5)
(SS) Good supervisor relationship	2.69 (0.62)	220 (28.6)	548 (71.4)
(SS) Supervisor listening support	2.66 (0.63)	243 (31.6)	525 (68.4)
(SS) Supervisor clarity about performance	2.71 (0.61)	216 (28.1)	552 (71.9)
(SS) Availability of work assistance	2.69 (0.59)	223 (29.0)	545 (71.0)
(SS) Workplace information communication	2.73 (0.57)	210 (27.3)	558 (72.7)
Social support at work	21.53 (3.65)		

JD: job demand, DL: decision latitude, SS: social support.

**Table 3:** Sociodemographic factors associated with psychosocial job characteristics and job strain among nurses in primary healthcare centers.

Characteristics	Psychosocial Job Demand		Decision Latitude (Job Control)		Social Support at Work		Job Strain				
	High/Very High	*p*	High/Very High	*p*	High/Very High	*p*	Low Strain n = 350, 45.6%	Active n = 326, 42.4%	Passive n = 44, 5.7%	High Strain n = 48, 6.3%	*p*
Sex											
Male	171 (81.0)	<0.001	182 (86.3)	0.354	167 (79.1)	0.001	32 (15.2)	150 (71.1)	8 (3.8)	21 (10.0)	<0.001
Female	203 (36.4)		494 (88.7)		492 (88.3)		318 (57.1)	176 (31.6)	36 (6.5)	27 (4.8)	
Age (year) *Mean (Standard Deviation)*	43.7 (6.5)	<0.001	40.7 (6.8)	0.608	40.1 (6.9)	<0.001	37.6 (5.7)	44.0 (6.2)	38.5 (7.5)	41.9 (8.0)	<0.001
Age category											
≤30	14 (23.3)	<0.001	47 (78.3)	0.124	57 (95.0)	<0.001	38 (63.3)	9 (15.0)	8 (13.3)	5 (8.3)	<0.001
31–40	110 (32.4)		304 (89.4)		310 (91.2)		212 (62.4)	92 (27.1)	18 (5.3)	18 (5.3)	
41–50	211 (65.1)		285 (88.0)		256 (79.0)		96 (29.6)	189 (58.3)	17 (5.2)	22 (6.8)	
>50	39 (88.6)		40 (90.9)		36 (81.8)		4 (9.1)	36 (81.8)	1 (2.3)	3 (6.8)	
Education level											
Vocational/College diploma	23 (29.1)	<0.001	50 (63.3)	<0.001	73 (92.4)	0.155	35 (44.3)	15 (19.0)	21 (26.6)	8 (10.1)	<0.001
Bachelor’s degree or postgraduate	339 (50.2)		617 (91.4)		573 (84.9)		313 (46.4)	304 (45.0)	23 (3.4)	35 (5.2)	
Other	12 (85.7)		9 (64.3)		13 (92.9)		2 (14.3)	7 (50.0)	0 (0)	5 (35.7)	
Marital status											
Married	196 (37.1)	<0.001	483 (91.3)	<0.001	463 (87.5)	0.007	312 (59.0)	171 (32.3)	21 (4.0)	25 (4.7)	<0.001
Single	101 (64.3)		122 (77.7)		135 (86.0)		33 (21.0)	89 (56.7)	23 (14.6)	12 (7.6)	
Other	77 (93.9)		71 (86.6)		61 (74.4)		5 (6.1)	66 (80.5)	0 (0)	11 (13.4)	
Housing status											
Own house	132 (86.8)	<0.001	128 (84.2)	0.262	115 (75.7)	<0.001	16 (10.5)	112 (73.7)	4 (2.6)	20 (13.2)	<0.001
Rented house	170 (32.0)		474 (89.1)		480 (90.2)		322 (60.5)	152 (28.6)	40 (7.5)	18 (3.4)	
Other	72 (85.7)		74 (88.1)		64 (76.2)		12 (14.3)	62 (73.8)	0 (0)	10 (11.9)	
Monthly income (million VND)											
<7 (~300 USD)	69 (42.3)	0.001	123 (75.5)	<0.001	150 (92.0)	0.017	67 (41.1)	56 (34.4)	27 (16.6)	13 (8.0)	<0.001
7–14 (300–600 USD)	296 (49.7)		544 (91.3)		502 (84.2)		283 (47.5)	261 (43.8)	17 (2.9)	35 (5.9)	
≥14 (≥600 USD)	9 (100)		9 (100)		7 (77.8)		0 (0)	9 (100)	0 (0)	0 (0)	
Caring for children under 5 years											
No	73 (28.1)	<0.001	218 (83.8)	0.011	244 (93.8)	<0.001	152 (58.5)	66 (25.4)	35 (13.5)	7 (2.7)	<0.001
Yes	301 (59.3)		458 (90.2)		415 (81.7)		198 (39.0)	260 (51.2)	9 (1.8)	41 (8.1)	
Caring for elderly or disabled family members											
No	57 (37.0)	0.001	121 (78.6)	<0.001	141 (91.6)	0.022	76 (49.4)	45 (29.2)	21 (13.6)	12 (7.8)	<0.001
Yes	317 (51.6)		555 (90.4)		518 (84.4)		274 (44.6)	281 (45.8)	23 (3.7)	36 (5.9)	

**Table 4:** Work-related characteristics associated with psychosocial job characteristics and job strain among nurses in primary healthcare centers.

Characteristics	Psychosocial Job Demand		Decision Latitude (Job Control)		Social Support at Work		Job Strain				
	High/Very High	*p*	High/Very High	*p*	High/Very High	*p*	Low Strain n = 350, 45.6%	Active n = 326, 42.4%	Passive n = 44, 5.7%	High Strain n = 48, 6.3%	*p*
Length of employment at healthcare centers (year)											
<3	10 (45.5)	<0.001	18 (81.8)	0.417	20 (90.9)	<0.001	12 (54.5)	6 (27.3)	0 (0)	4 (18.2)	<0.001
3–6	27 (23.1)		99 (84.6)		111 (94.9)		76 (65.0)	23 (19.7)	14 (12.0)	4 (3.4)	
7–9	85 (29.5)		256 (88.9)		257 (89.2)		183 (63.5)	73 (25.3)	20 (6.9)	12 (4.2)	
>9	252 (73.9)		303 (88.9)		271 (79.5)		79 (23.2)	224 (65.7)	10 (2.9)	28 (8.2)	
Current working department											
Clinical departments	148 (27.9)	<0.001	468 (88.3)	0.038	473 (89.2)	<0.001	340 (64.2)	128 (24.2)	42 (7.9)	20 (3.8)	<0.001
Paraclinical departments	82 (95.3)		78 (90.7)		69 (80.2)		4 (4.7)	74 (86.0)	0 (0)	8 (9.3)	
Administrative departments	127 (94.8)		118 (88.1)		109 (81.3)		5 (3.7)	113 (84.3)	2 (1.5)	14 (10.4)	
Logistics/support departments	17 (94.4)		12 (66.7)		8 (44.4)		1 (5.6)	11 (61.1)	0 (0)	6 (33.3)	
Weekly working hours											
≤40	14 (3.7)	<0.001	337 (89.2)	0.610	378 (100)	<0.001	325 (86.0)	12 (3.2)	39 (10.3)	2 (0.5)	<0.001
41–48	61 (85.9)		61 (85.9)		44 (62.0)		9 (12.7)	52 (73.2)	1 (1.4)	9 (12.7)	
>48	299 (93.7)		278 (87.1)		237 (74.3)		16 (5.0)	262 (82.1)	4 (1.3)	37 (11.6)	
Holding managerial or additional positions											
No	135 (26.8)	<0.001	445 (88.5)	0.598	471 (93.6)	<0.001	327 (65.0)	118 (23.5)	41 (8.2)	17 (3.4)	<0.001
Yes	239 (90.2)		231 (87.2)		188 (70.9)		23 (8.7)	208 (78.5)	3 (1.1)	31 (11.7)	

## Data Availability

The data presented in this study are available upon request from the corresponding author.
